# Transcrestal Sinus Floor Elevation and Simultaneous Implant Placement in a Severely Atrophic Maxillary Ridge: A Case Report

**DOI:** 10.1002/ccr3.73375

**Published:** 2026-08-20

**Authors:** Luca Ortensi, Raffaele Mirra, Tommaso Vitali, Aroldo Zoli, Armando Taccone, Francesco Saverio Canova

**Affiliations:** ^1^ Private Practice Bologna Italy

**Keywords:** atrophic maxilla, dental implants, oral surgery, sinus floor augmentation

## Abstract

Carefully selected patients with severe posterior maxillary atrophy may be successfully treated using a modified transcrestal sinus floor elevation. An inwardly displaced crestal bony window may facilitate membrane elevation while allowing simultaneous implant placement with minimal surgical invasiveness.

## 1 Introduction

The maxillary sinus augmentation procedure was firstly described by Tatum in the 1970s; however, Boyne and James published the detailed description of the technique in 1980 [1]. Since then, several researchers and clinicians contributed to improve this procedure, focusing on physiology, surgical technique and biomaterials. Different grafting materials, including autogenous bone, xenografts, alloplastic substitutes, and more recently graftless approaches, have been investigated to optimize bone regeneration and clinical outcomes [2, 3]. Nowadays, sinus lift is a well‐established procedure with documented long‐term efficacy. However, postoperative morbidity and the overall duration of sinus augmentation protocols remain subjects of extensive discussion, with modern research shifting toward minimizing patient discomfort and treatment timelines. Summers in 1994 described the crestal approach to sinus augmentation conducted by means of osteotomes [4], however, this procedure can guarantee a sufficient success rate only when residual bone height (RBH) is at least 5 mm [5]. Furthermore, the use of manual osteotomes carries an inherent risk of benign paroxysmal positional vertigo, with a reported incidence of 5.84% [6]. The morbidity of the transcrestal approach appears to be higher than the lateral approach the first day after surgery [7, 8], thus, multiple modifications to the sinus augmentation procedure have been introduced to minimize complication rates and improve patient comfort [9, 10, 11, 12, 13]. Flap design, lateral window dimension, different devices for site preparation and sinus floor elevation were examined, despite some gray zones persisting regarding the precise indications for each variation, since other factors, such as operator skills and anatomical variability, may play significant roles. Indeed, transcrestal sinus floor elevation, despite being easier to handle by the clinician, may raise the risk of perforation in large maxillary sinuses [14]. Recently, Stacchi et al. developed a decision tree which overcomes previous classifications based on the sole residual bone height [15], integrating it with horizontal sinus width [16]. In this context, the biologic principles underlying bone regeneration should be emphasized as summarized by Wang et al. [17]. Subantral space represents an ideal environment to get primary wound coverage, space maintenance, wound stability and adequate blood supply. Nonetheless, there has been a long debate on the active role of the Schneiderian membrane on the regeneration process, which finally resulted less involved than expectations. In fact, blood supply and osteogenic stimuli depend on the amount of denuded sinus walls [18].

Given the aforementioned point, with the present case report the authors want to merge the principle of minimal invasiveness and respect for biologic principles in order to obtain a sinus augmentation procedure that allows a reduced extension of the flap and favors an adequate sinus membrane elevation.

## 2 Case History

### 2.1 Patient Characteristics

A 56‐year‐old Caucasian man referred to the private dental practice complaining of reduced masticatory efficiency due to pain during the chewing cycle on the left side. The patient was in good general health and reported no systemic diseases or contraindications to dental surgery. The dental history was collected and the only treatment the patient has ever been subjected to was professional mechanical plaque removal and the prosthetic treatment of upper‐left molars due to caries. Clinical examination revealed previously undiagnosed periodontitis Stage III grade C and teeth 2.6 and 2.7 with severe attachment loss, Grade 3 mobility [19] and Grade 3 furcation involvement [20] (Figure 1). No other hard or soft tissue pathologies were identified, and the remaining components of the stomatognathic system appeared healthy. Previously restored teeth and existing dental treatments were clinically stable, with no evidence of recurrent disease or treatment failure.

**FIGURE 1:**
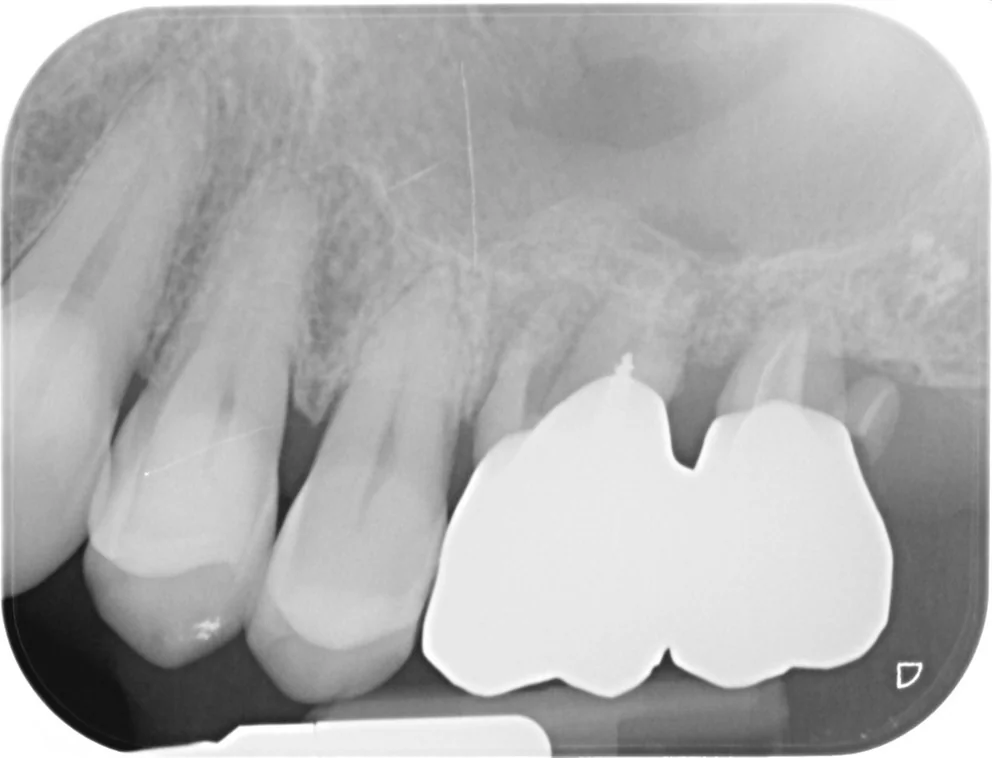
Intraoral radiograph of upper left molars at baseline.

The chief complaint was the resolution of masticatory dysfunction.

### 2.2 Preliminary Treatment

Firstly, active periodontal therapy was conducted. The treatment comprised the control of risk factors for periodontitis, removal of plaque and calculus, and the extraction of teeth judged irrational to treat (i.e., 2.6 and 2.7) in accordance with the European Federation of Periodontology (EFP) S3‐level clinical practice guidelines for the prevention of peri‐implant diseases [21, 22]. Three months after extraction, a reevaluation was performed, and an intraoral radiograph of the third sextant was obtained to assess bone healing. The edentulous ridge appeared fully healed (Figure 2) and radiographic examination revealed uniform residual bone and the absence of lamina dura, indicating an advanced healing process at the extraction sites.

**FIGURE 2:**
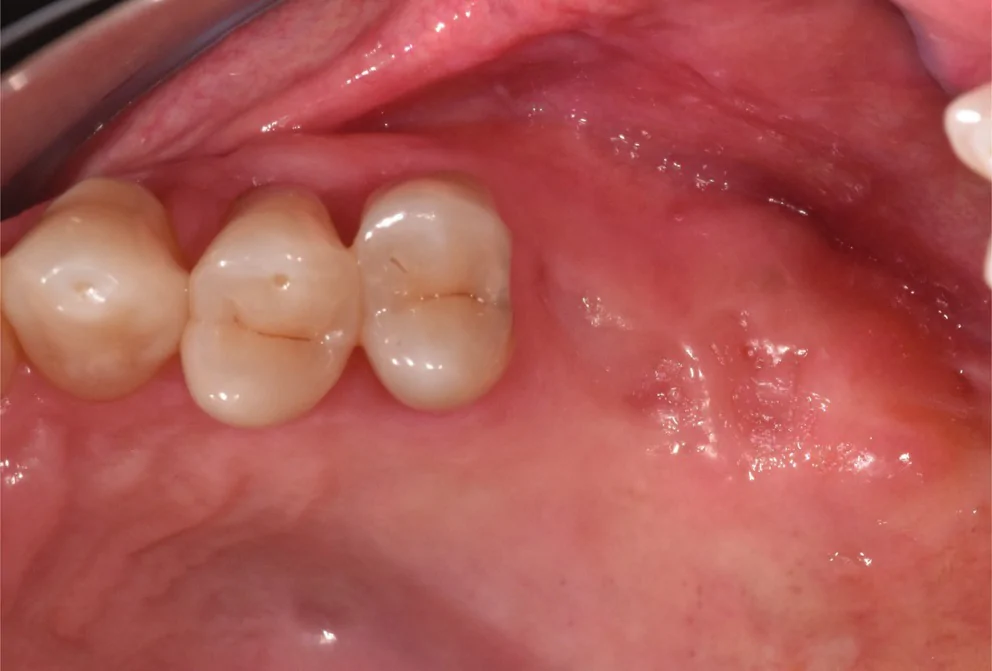
Clinical picture of fully healed alveolar ridge 3 months after extractions.

### 2.3 Diagnostic Records

Because the RBH appeared to be approximately 3 mm on the intraoral radiograph, advanced diagnostic records were collected to establish an accurate, predictable treatment plan. Intraoral digital scans and Cone Beam Computed Tomography (CBCT) were acquired. Based on the radiographic findings, a Class C defect according to the classification of Chiapasco et al. was diagnosed, characterized by a RBH less than 4 mm, a residual alveolar ridge width of 5 mm, and an acceptable vertical intermaxillary relationship [23] (Figure 3).

**FIGURE 3:**
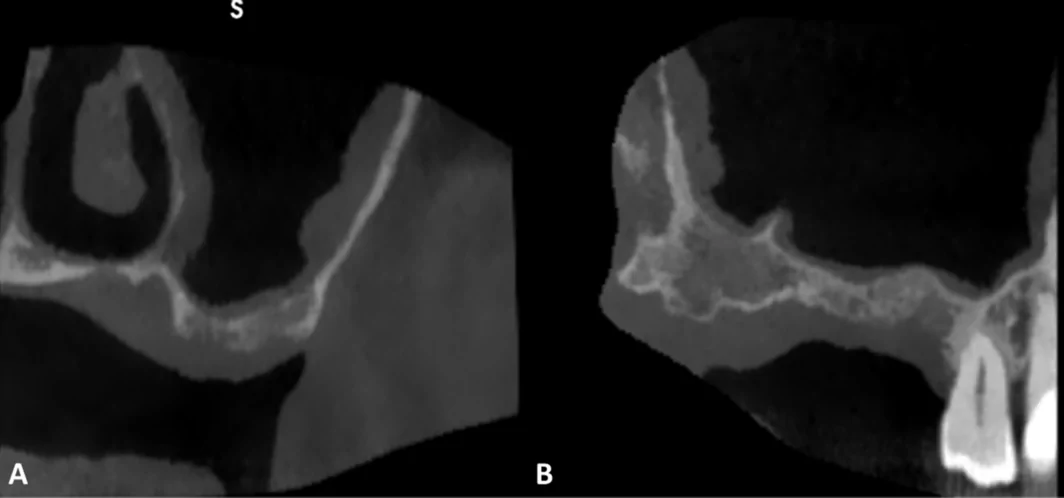
Pre‐operative CBCT illustrating transversal (A) and sagittal (B) sections of residual alveolar ridge 3 months after extractions.

### 2.4 Crestal Approach for Sinus Augmentation and Implant Placement

Following the case discussion, a one‐stage surgical approach was selected, planning simultaneous implant placement and sinus elevation. As reported by Stacchi et al., a conventional transcrestal sinus lift would have carried a significant risk of membrane perforation due to the maxillary sinus' transverse width exceeding 12 mm [16]. Therefore, a modified technique was implemented.

Prophylactic antibiotic therapy consisting of 2 g of amoxicillin was administered 30 min prior to the procedure. Following a 1‐min pre‐operative rinse with 0.2% chlorhexidine diglucanate, local infiltration anesthesia (4% articaine with 1:100,000 epinephrine) was delivered into the buccal alveolar mucosa of the edentulous site and palatally. Using a 15C blade, an intrasulcular incision was made at the second premolar and extended midcrestally from the mid‐distal aspect of the tooth to the maxillary tuberosity. A full‐thickness mucoperiosteal flap was elevated to expose the residual crest.

A piezoelectric handpiece equipped with a dedicated bone‐cutting tip (Piezosurgery, Mectron, Carasco, Italy) was utilized to outline a rectangular slot centered directly over the crest, encompassing both planned implant sites (Figure 4). The window was designed to extend 2 mm mesially and distally beyond the planned implant positions, while its width was intentionally kept 2 mm narrower than the planned implant diameter (3 mm width in this specific case) (Figure 5). The osteotomy was carried through the residual crestal bone up to the sinus floor (preserving its integrity) with light pressure and continuous sterile saline irrigation to prevent overheating and avoid damaging the underlying Schneiderian membrane.

**FIGURE 4:**
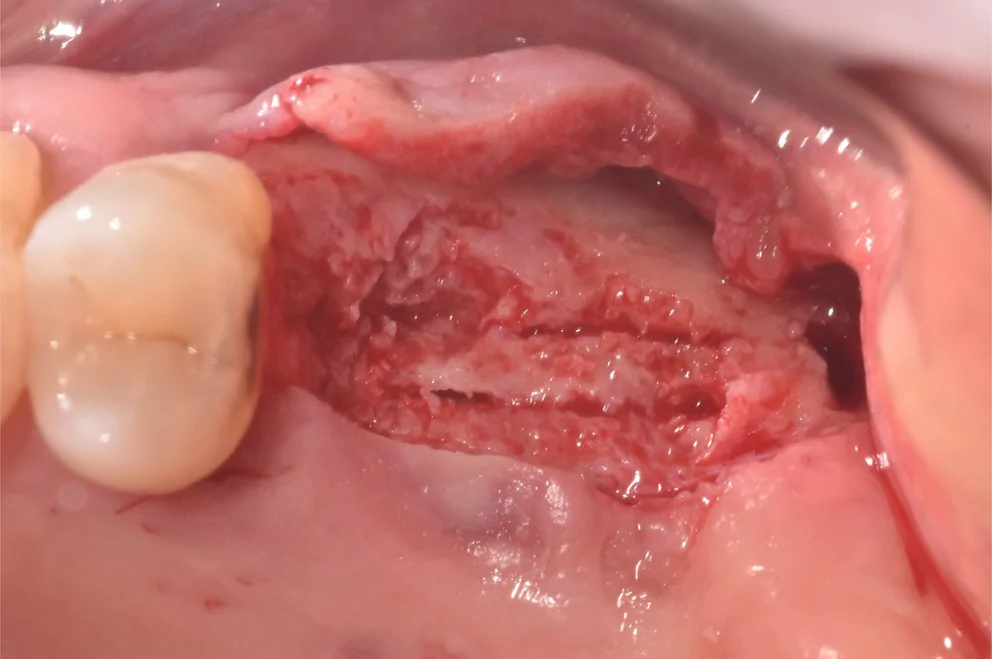
Intraoperative clinical view showing the precise rectangular crestal osteotomy performed with a piezoelectric device over the residual ridge.

**FIGURE 5:**
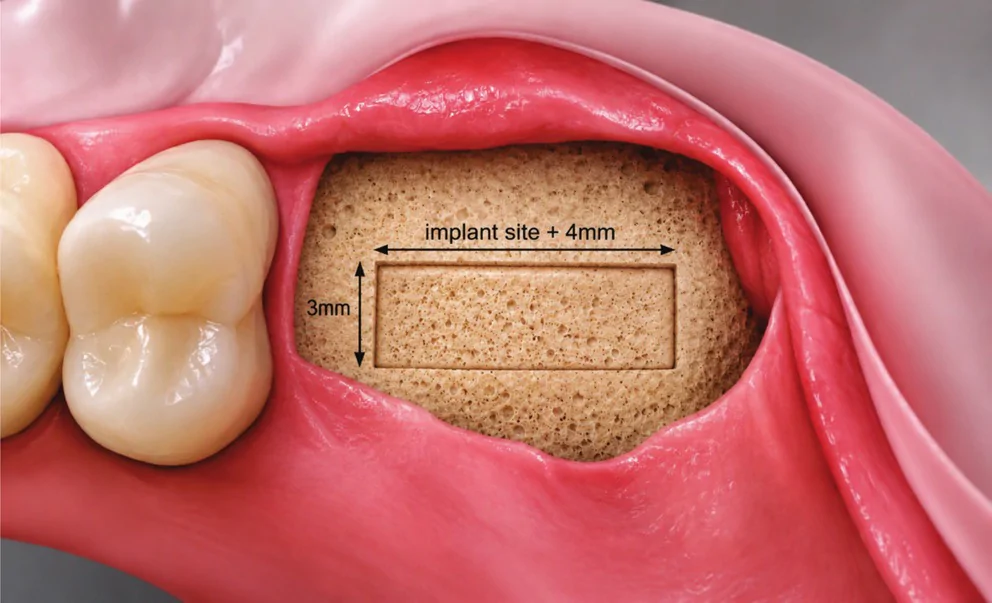
Schematic diagram illustrating the proportional design of the crestal bony window relative to the final implant diameters.

Once the osteotomy was completed, the rectangular bony segment was gently displaced cranially using an osteotome, creating a greenstick fracture of the sinus floor while maintaining its attachment to the Schneiderian membrane. After sufficient access to the subantral space had been achieved, the Schneiderian membrane was carefully elevated using dedicated sinus membrane elevators. Following adequate elevation of the membrane and exposure of the sinus floor from the medial to the lateral wall, the implant osteotomies were prepared according to the manufacturer's recommended drilling protocol.

Two bone‐level dental implants of 5 mm in diameter and 8 mm in length (Nevo, Thommen Medical AG; Grenchen, CH) were inserted into the prepared sites (Figure 6). Primary stability values were 25 Ncm for the implant in 2.6 site and 20 Ncm for the one in 2.7 position. The cranial displacement of the bony window was maintained by the implant apices, simultaneously protecting the Schneiderian membrane and providing an osteogenic surface distant from the sinus walls.

**FIGURE 6:**
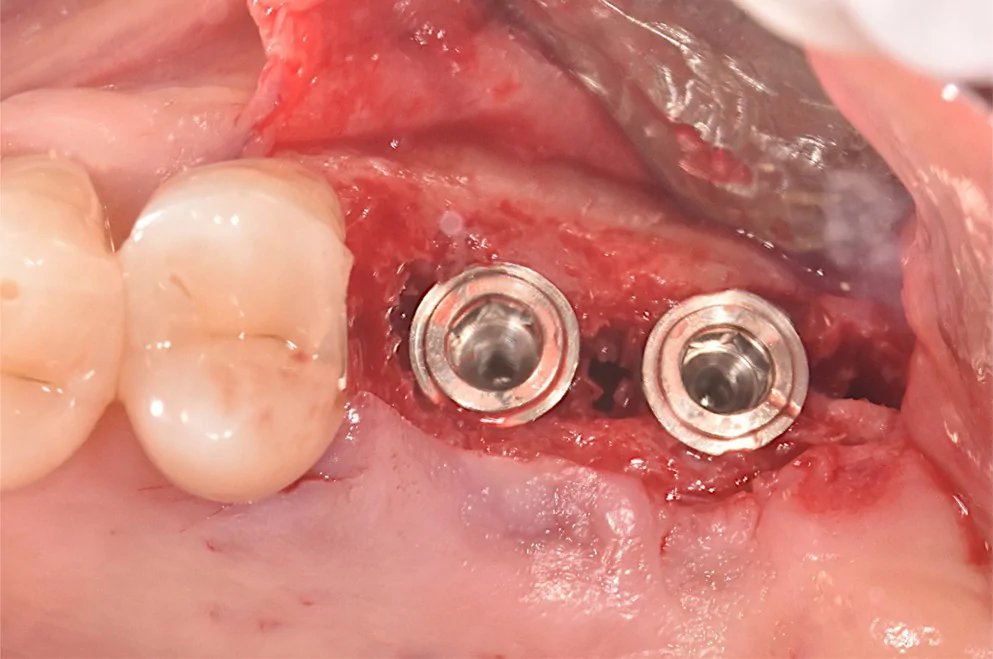
Intrasurgical view of implant placement.

A submerged healing protocol was planned. The mucoperiosteal flap was repositioned and sutured using horizontal mattress sutures alternated with single interrupted sutures. Postoperative antibiotic and analgesic therapy was administered. Sutures were removed 2 weeks after surgery.

### 2.5 Implant Exposure and Prosthetic Phases

Five months after healing, a second‐stage surgery was performed to expose the submerged implants (Figure 7). Healing abutments were connected, and interrupted sutures were placed to stabilize the flap. One month later, the implant positions were recorded by means of an intraoral scan (Figure 8). The prosthetic workflow consisted of the verification of the metal framework, the ceramic try‐in, and ultimately the delivery of the definitive prosthesis. The implant screws were tightened to the torque value recommended by the manufacturer.

**FIGURE 7:**
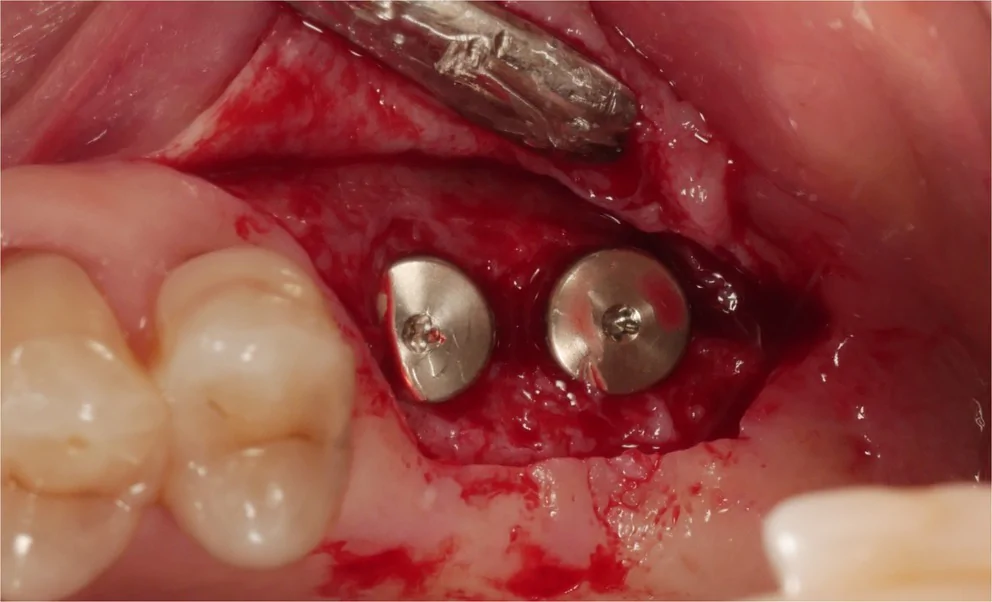
Clinical view at the 5‐month second‐stage surgery during the connection of the transmucosal healing abutments.

**FIGURE 8:**
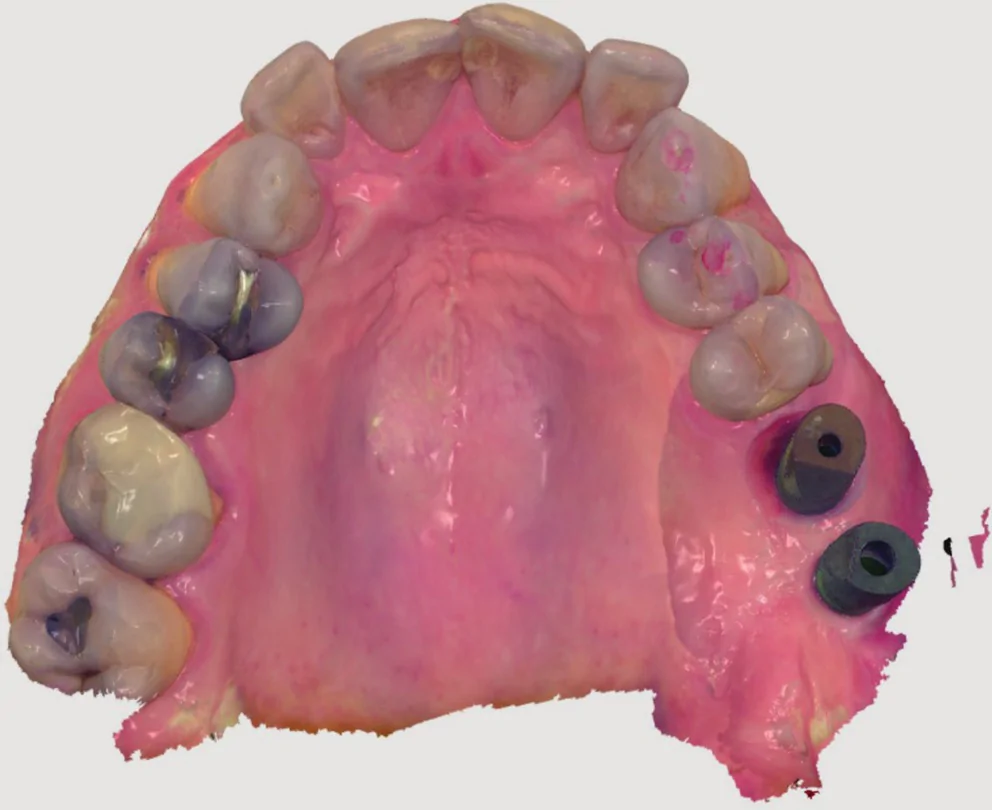
Digital intraoral scan utilized for the precise CAD/CAM fabrication of the final screw‐retained prosthetic framework.

### 2.6 Follow‐Up

At the 12‐month follow‐up, no mechanical or biological complications were detected (Figure 9). Clinical examination showed physiologic probing depths along the transmucosal pathway, with an absence of bleeding on probing (Figure 10). Access for oral hygiene procedures was adequate, and the patient reported no episodes of food impaction.

**FIGURE 9:**
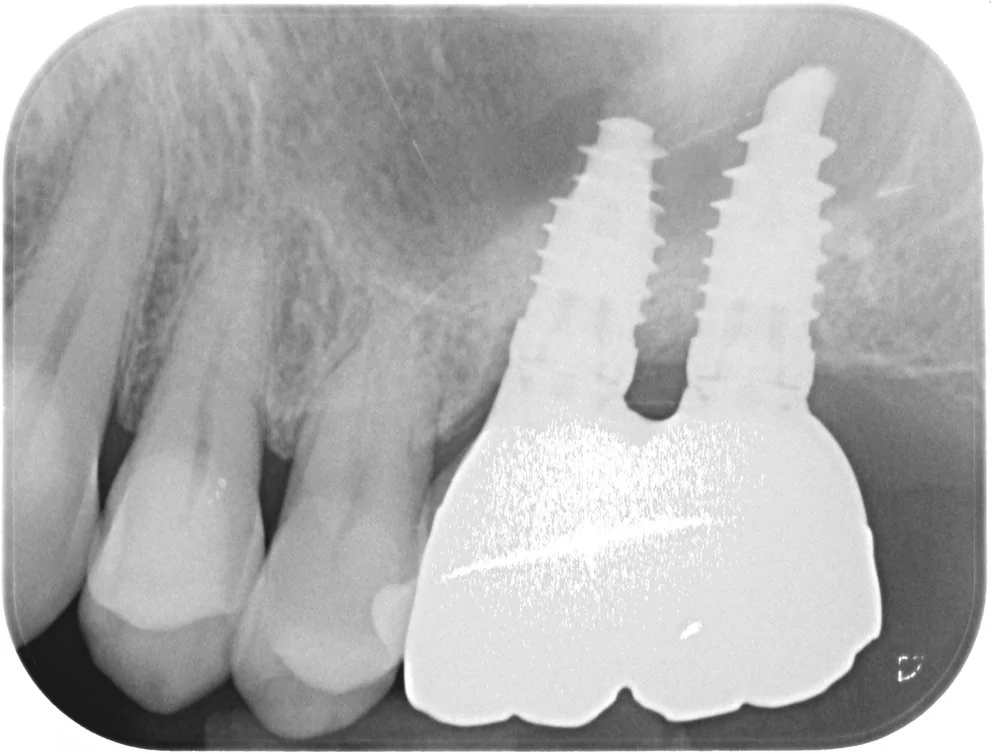
Post‐operative periapical radiograph at the 12‐month follow‐up, showing stable peri‐implant bone levels and successful subantral bone consolidation.

**FIGURE 10:**
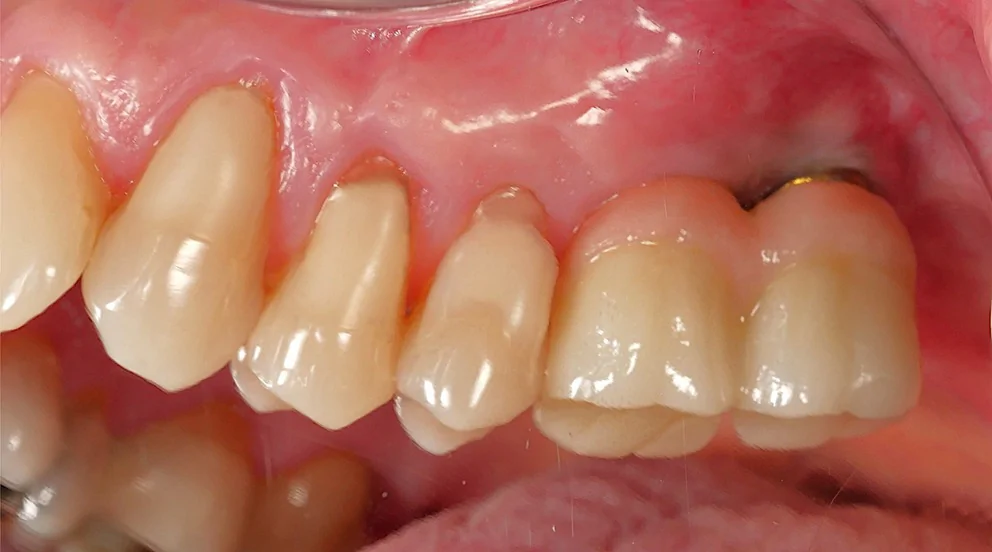
Definitive clinical view of the screw‐retained ceramic prosthesis delivered to the patient.

## 3 Discussion

The present case report describes a novel variation of sinus augmentation technique designed to maximize the technical and biological advantages of both crestal and lateral approaches. Structurally, this technique allows a smaller surgical wound than the lateral window technique; biologically, it may enhance the osteogenic potential of the subantral space, maximizing the bony walls involved in the healing process.

Preclinical studies showed the influence of the antrostomy in the lateral approach for sinus augmentation. The absence of the bony wall, whether or not a collagen membrane in positioned over the window, affect the amount of new bone formation during the healing process [24, 25]. Moreover, Amari et al. in a study conducted on rabbits, showed that the reflection of the bony wall on the roof of the subantral space, can improve the total amount of bone after 8 weeks of healing [26]. We can therefore speculate that the crestal bone dislocated apical to the implants could theoretically promote a faster and more even regeneration, however the same authors also underline that due to the dimensional differences between rabbit and human, the proportion of the reflected bone on the total regenerative space may weaken the result in human.

Furthermore, the present approach may facilitate a progressive elevation of the sinus membrane, possibly favoring greater control over its integrity, thus balancing the higher rate of membrane perforation associated with conventional transcrestal technique when vertical augmentation is required in more than 3 mm [27]. In fact, a properly planned bony window brings two benefits: first, a good access for the membrane elevators to the subantral space, and additionally provides close bony edges in order to reach a favorable primary stability of the fixture. In this context, careful preoperative planning is crucial to ensure appropriate implant selection, providing adequate primary stability while maintaining sufficient buccal bone thickness, with the implant diameter exceeding the width of the bony window by approximately 2 mm.

Despite its strengths, this procedure presents some limitations. In fact, the surgeon must have a thorough understanding of the implant system so as to perform an adequate under‐preparation of the site and avoid excessive loss of primary stability, potentially requiring a wider implant or a two‐stage protocol if the implant cannot be stabilized. Moreover, due to the narrow space of the bony window, an excessive RBH may hamper the detachment maneuvers despite the finest instruments employed.

## 4 Conclusion

The proposed technique fits within a broad spectrum of clinical scenarios in which the patient's individual anatomical characteristics guide the decision‐making process. Particularly, the present technique may be considered in cases presenting with a sinus with a wide transverse dimension. The balance between risks and benefits associated with each approach must be integrated with the clinician's expertise to ensure the best possible outcome for the patient. Recent literature increasingly highlights the importance of personalized treatment, and any modification of the conventional technique should be validated to confirm its actual clinical benefit and contribute to shared scientific knowledge. Further clinical studies are warranted to validate this approach before widespread clinical adoption.

## Author Contributions

**Tommaso Vitali:** methodology. **Aroldo Zoli:** writing – review and editing. **Luca Ortensi:** conceptualization, investigation. **Francesco Saverio Canova:** writing – review and editing. **Armando Taccone:** visualization. **Raffaele Mirra:** conceptualization, writing – original draft.

## Funding

The authors have nothing to report.

## Consent

Written informed consent was obtained from the patient for publication of this case report and accompanying images.

## Conflicts of Interest

The authors declare no conflicts of interest.

## Data Availability

Deidentified data supporting the findings of this case report are available from the corresponding author upon reasonable request and subject to patient confidentiality and institutional restrictions.
